# Effect of insulin levels and plasma fasting glucose on refractive status in the United States population aged 12–19 years

**DOI:** 10.3389/fmed.2023.1269671

**Published:** 2023-11-24

**Authors:** Pengcheng Hu, Jialing Liu, Ming He, Yuxian Fu, Menglei Wang

**Affiliations:** ^1^Department of Ophthalmology, The Second Affiliated Hospital of Chongqing Medical University, Chongqing Medical University, Chongqing, China; ^2^Department of Phase I Clinical Trial Center, The Second Affiliated Hospital of Chongqing Medical University, Chongqing Medical University, Chongqing, China

**Keywords:** insulin levels, plasma fasting glucose, myopia, NHANES, adolescents

## Abstract

**Purpose:**

The causes of myopia are varied, and both genetic and environmental influences play an essential role. The prevalence of myopia is increasing among adolescents and is expected to be more than one-third the global population by 2050. Some animal studies suggest that hyperinsulinemia may be a cause of myopia. In this context, the purpose of this paper is to investigate the potential effects of insulin levels and plasma fasting glucose on the refractive status of adolescents.

**Methods:**

Data were collected from the National Health and Nutrition Examination Survey (NHANES) from 1999 to 2008. Weighted multivariable linear regression models were used to assess the effect of insulin levels, plasma fasting glucose on refractive status. We used a smooth curve fit to reveal the nonlinear relationship between the variables.

**Results:**

In the multivariate regression model, as insulin levels increased, there was a shift towards myopia in refractive status (*β* = −0.013, 95% CI: −0.023 to −0.004). This correlation was also evident in the male adolescent subgroup (*β* = −0.021, 95% CI: −0.035 to −0.007). Similar findings indicated that in Mexican Americans, there was a myopic shift in refractive status as insulin levels increased (*β* = −0.018, 95% CI: −0.033 to −0.002). However, grouping by differences in insulin measurement showed no significant correlation in Mexican-Americans. At the same time, there was no significant correlation between plasma fasting glucose and refractive status (*β* = −0.041, 95% CI: −0.174 to 0.091).

**Conclusion:**

The present cross-sectional study demonstrated that higher insulin levels may promote the development of myopia in adolescents, but there may be variations across gender and ethnicity. More basic research is needed to reveal the mechanistic reasons for the association.

## Introduction

Myopia has become a global public health problem, and its incidence is growing year by year, seriously affecting the vision and visual quality of teenagers (1). It is well recognized that genetics plays an essential role in the development of myopia, but genes cannot be blamed for all myopia occurrences (2, 3). With the prevalence of electronic products and the emphasis on education, there has been a significant increase in close work time for adolescents, which may be one of the risk factors for myopia (4). In the meantime, less outdoor activity can also contribute to the development of myopia (5, 6). Another factor associated with myopia is consumption of refined carbohydrates, where prolonged high sugar intake has a significant effect on the body’s serum glucose and insulin levels (7, 8).

As the human economy improves, high-carbohydrate diets and high sugar intake become more common. The eye axis of adolescents in the developmental stage is also growing, and insulin is associated with eye axis growth in animal studies (9). It has also been suggested that a high glycemic load may increase the incidence of myopia, but the outcomes of the current researches are not entirely consistent. For this reason, we conducted a cross-sectional study with a larger sample based on the entire U.S. population to determine the relationship between insulin levels, plasma fasting glucose, and adolescent refractive status. Simultaneously, we conducted an in-depth examination of the disparities across different genders and ethnicities, with the goal of devising customized testing protocols suitable for distinct population cohorts.

## Methods

### Study design

The National Health and Nutrition Examination Survey (NHANES) is a cross-sectional survey based on the health of the United States population. The survey, which began in 1960, is conducted on approximately 5,000 residents each year using a complicated multi-stage probability sampling design. The main content of the survey includes demographic data, dietary data, laboratory biochemical data, and hundreds of other indicators. Some years also include additional examination data, such as vision exams related to ophthalmology. The project was endorsed by the National Center for Health Statistics (NCHS) Ethics Review Committee, and written informed consent was received from each participant (aged ≥18 years) or their parents/guardians (aged <18 years) (10, 11). At the same time, this study observed the principles of the Declaration of Helsinki.

### Study participants

We used data from NHANES for a total of 5 cycles from 1999–2008, because refractive status was collected only during this period. We included participants aged 12–19 years, because NHANES only covered the refractive status of participants aged 12 years or older. The upper age limit of 19 years was set to observe the effect of fasting glucose and insulin on refractive status in the developmental stage. A total of 4,305 subjects contained complete refractive status results, fasting glucose and insulin levels. Exclusion criteria for participants were: previous myopic refractive surgery or cataract surgery (*n* = 6), unclear history of ocular surgery (*n* = 8), using insulin (*n* = 16), and taking oral hypoglycemic agents (*n* = 3), 4,272 subjects eventually met the requirements and were included in this study.

### Variables

The independent variables of this study were serum insulin level and plasma fasting glucose. Serum insulin concentrations were measured by enzyme immunoassay from 2003 to 2008 and Insulin radioimmunoassay from 1999 to 2002. Because of the difference in insulin measurement methods, we conducted separate analyses for the data from these two stages. Plasma fasting glucose was calculated using the hexokinase method.

We calculated the spherical equivalent (half of the cylinder plus the sphere) to measure the patient’s refractive status. The refractive status of the participants was calculated by means of an autorefractor (Nidek ARK-760; NIDEK Co., Ltd., Tokyo, Japan). Since there was no paralysis of the ciliary muscle and there may be stronger accommodation in younger participants, the effect of this aspect on refractive status was minimized by the function of auto fogging. The eyes included in the analysis were the participants’ right eyes, and the refractive error analyzed was the median of three objective optometric measurements.

Age, gender, race, education level, and income poverty ratio (PIR) were controlled for in the statistical analysis as demographic covariates, in order to rule out potential confounding. These data could be obtained in demographics data. Total serum calcium and glycohemoglobin were extracted from laboratory data. The procedure for acquiring serum insulin, plasma fasting glucose, refractive status and other covariates can be found on the NHANES dataset.^1^

### Statistical analysis

All statistical analysis of the data was implemented using R^2^ and EmpowerStats software.^3^ Continuous variables were summarised as median [interquartile range (IQR)]. Categorical variables were summarised as percentages [95% confidence interval (CI)]. Weighted linear regression models were utilized for continuous variables, and weighted chi-square tests were utilized for categorical variables, to assess disparities between different groups. When the value of *p* was less than 0.05, we considered a statistically significant difference. According to the NCHS analysis guidelines, sample weights were used in the calculation of the statistical analysis data. We explained the potential relationship between the variables using multivariable linear regression models. Since there were two independent variables, serum insulin and plasma fasting glucose, we performed a subgroup analysis by gender and race. Additionally, we conducted a trend test to examine the linear trend of insulin levels and plasma fasting glucose with refractive status. When the P for trend was less than 0.05, we considered this linear trend to be significant. For the nonlinear relationship between variables, we also conducted generalized additive model and spline smoothing plot to solve it.

## Results

The basic characteristics of the included participants were displayed in Table 1. There were 2,234 male participants, representing 52% of the total. The participants’ refractive status ranged from myopia of −20.25 D to hyperopia of +9.5 D, and the median [interquartile range (IQR)] spherical equivalent was −0.250 (−1.375, 0.125) D. The median insulin levels [interquartile range (IQR)] were 56.340 (39.480, 81.720) pmol/L for males and 64.380 (46.740, 89.880) pmol/L for females. Median fasting serum glucose concentrations [interquartile range (IQR)] were 5.218 (4.940–5.495) mmol/L and 4.994 (4.752–5.273) mmol/L for male and female participants, respectively.

**Table 1:** The demographic and laboratory data of the participants: a cross-sectional study using data from NHANES 1999–2008 (*N* = 4,272).

Characteristics	Female (*n* = 2038)	Male (*n* = 2,234)	Value of *p*
Age (%)			0.782
12–15 years	48.810 (45.280,52.340)	49.470 (46.242,52.699)	
16–19 years	51.190 (47.660,54.720)	50.530 (47.301,53.758)	
Race (%)			0.817
Non-Hispanic White	62.520 (58.055,66.985)	61.428 (57.522,65.334)	
Non-Hispanic Black	14.286 (11.754,16.819)	15.143 (12.618,17.669)	
Mexican American	11.252 (8.843,13.661)	11.074 (9.111,13.037)	
Other Hispanic	6.034 (4.186,7.882)	5.629 (3.815,7.444)	
Other Race	5.908 (3.930,7.885)	6.726 (4.582,8.870)	
Education level (%)			0.775
Less Than 9th Grade	42.514 (39.187,45.841)	43.109 (40.230,45.989)	
9th Grade or higher	57.486 (54.159,60.813)	56.891 (54.011,59.770)	
Income poverty ratio, median (IQR)	2.380 (1.060,4.110)	2.360 (1.130,4.100)	0.890
Spherical equivalent, median (IQR), D	−0.250 (−1.500,0.125)	−0.250 (−1.250,0.125)	0.145
Insulin levels, median (IQR), pmol/L	64.380 (46.740,89.880)	56.340 (39.480,81.720)	<0.001
Plasma Fasting Glucose, median (IQR), mmol/L	4.994 (4.752,5.273)	5.218 (4.940,5.495)	<0.001
Glycohemoglobin, median (IQR), (%)	5.100 (4.900,5.300)	5.100 (5.000,5.300)	<0.001
Total calcium, median (IQR), mmol/L	2.400 (2.350,2.450)	2.450 (2.400,2.475)	<0.001

Continuous variables were expressed as median [interquartile range (IQR)]. Categorical variables were expressed as percentages [95% confidence interval (CI)]. Student’s *t*-test and Wilcoxon test were used for continuous variables, and chi-square test was used for categorical variables.

In comparison to female participants, men had markedly lower levels of serum insulin, and had higher levels of plasma fasting glucose, glycohemoglobin, and total calcium. No significant differences were seen in age, race, education level, and income poverty ratio.

Table 2 demonstrated the relationship between serum insulin concentration and refractive status in the different multivariable linear regression models and stratified analyses. It was shown that higher serum insulin levels were associated with more negative refractive status. This negative correlation was more significant in men (*β* = −0.021, 95% CI: −0.035 to −0.007) with each increase of 10 pmol/L. This situation was also shown in Mexican American (*β* = −0.018, 95% CI: −0.033 to −0.002). There was no significant negative correlation between females, non-Hispanic white, non-Hispanic Black, other Hispanic and other races. In different quartiles of insulin, this negative correlation was found in Q3 (*β* = −0.234, 95% CI: −0.398 to −0.070) and Q4 (*β* = −0.284, 95% CI: −0.459 to −0.109). P for trend was less than 0.05.

**Table 2:** The association between insulin (10 pmol/L) and spherical equivalent (D).

	Non-adjusted, *β* (95% CI)	Adjust I, *β* (95% CI)	Adjust II, *β* (95% CI)
Insulin (10 pmol/L)	−0.010 (−0.019, −0.001)	−0.011 (−0.020, −0.002)	−0.013 (−0.023, −0.004)
Stratified by gender			
Male	−0.018 (−0.031, −0.005)	−0.020 (−0.033, −0.007)	−0.021 (−0.035, −0.007)
Female	−0.002 (−0.014, 0.011)	−0.002 (−0.015, 0.010)	−0.005 (−0.019, 0.009)
Stratified by race			
Mexican American	−0.023 (−0.037, −0.008)	−0.022 (−0.036, −0.007)	−0.018 (−0.033, −0.002)
Other Hispanic	0.002 (−0.036, 0.040)	0.002 (−0.037, 0.040)	−0.004 (−0.047, 0.040)
Non-Hispanic White	−0.015 (−0.035, 0.006)	−0.016 (−0.037, 0.005)	−0.018 (−0.040, 0.003)
Non-Hispanic Black	−0.001 (−0.012, 0.011)	0.000 (−0.012, 0.012)	−0.002 (−0.016, 0.012)
Other Race	−0.007 (−0.044, 0.029)	−0.007 (−0.044, 0.029)	−0.008 (−0.047, 0.030)
Quintiles of Insulin			
Lowest quintile (4.26–43.80 pmol/L)	Reference	Reference	Reference
Q2 (43.86–63.30 pmol/L)	−0.110 (−0.262, 0.041)	−0.106 (−0.257, 0.046)	−0.145 (−0.304, 0.014)
Q3 (63.36–94.98 pmol/L)	−0.199 (−0.354, −0.044)	−0.203 (−0.359, −0.047)	−0.234 (−0.398, −0.070)
Q4 (95.04–1387.44 pmol/L)	−0.221 (−0.383, −0.058)	−0.243 (−0.408, −0.079)	−0.284 (−0.459, −0.109)
*p* for trend	0.003	0.001	<0.001

Non-adjusted model adjust for: none. Adjust I model adjust for: gender, age, race. Adjust II model adjust for: gender, age, race, education level, income poverty ratio, calcium, glycohemoglobin.

To minimize the deviations, we divided the data from 1999 to 2002 into one group and the data from 2003 to 2008 into another group due to differences in methods of insulin determination. The results were presented in Table 3 and Table 4, respectively. After adjusting for covariates between serum insulin and spherical equivalent, each 10 pmol/L insulin level increase was associated with *β* −0.016, 95% confidence interval (CI) −0.031 to −0.002 in 1999–2002 and *β* −0.013, 95% confidence interval (CI) −0.026 to 0.001 in 2003–2008. The results continued to suggest a negative correlation between insulin levels and spherical equivalent. In the male subgroup, the negative correlation trend was still significant, with *β* values of −0.027 (95% CI: −0.049 to −0.005) and −0.018 (95% CI: −0.036 to −0.000) respectively. In Table 3, we observed a consistent negative correlation in quartiles Q2 (*β* = −0.404, 95% CI: −0.629 to −0.179), Q3 (*β* = −0.403, 95% CI: −0.635 to −0.170), and Q4 (*β* = −0.256, 95% CI: −0.506 to −0.006) compared to Q1 when insulin levels were stratified. In Table 4, a similar pattern was observed in Q4 (*β* = −0.308, 95% CI: −0.552 to −0.064). This trend remained robust and statistically significant (*p* for trend <0.05).

**Table 3:** The association between insulin (10 pmol/L) and spherical equivalent (D) in 1999–2002.

	Non-adjusted, *β* (95% CI)	Adjust I, *β* (95% CI)	Adjust II, *β* (95% CI)
Insulin (10 pmol/L)	−0.018 (−0.032, −0.005)	−0.017 (−0.031, −0.004)	−0.016 (−0.031, −0.002)
Stratified by gender			
Male	−0.033 (−0.053, −0.012)	−0.031 (−0.051, −0.010)	−0.027 (−0.049, −0.005)
Female	−0.006 (−0.023, 0.010)	−0.007 (−0.024, 0.010)	−0.007 (−0.026, 0.012)
Stratified by race			
Mexican American	−0.007 (−0.026, 0.012)	−0.007 (−0.026, 0.012)	−0.003 (−0.024, 0.018)
Other Hispanic	−0.005 (−0.081, 0.070)	0.012 (−0.065, 0.089)	0.027 (−0.089, 0.143)
Non-Hispanic White	−0.021 (−0.055, 0.013)	−0.022 (−0.055, 0.012)	−0.018 (−0.053, 0.018)
Non-Hispanic Black	−0.008 (−0.027, 0.011)	−0.007 (−0.026, 0.013)	−0.006 (−0.027, 0.016)
Other Race	−0.026 (−0.058, 0.007)	−0.032 (−0.062, −0.001)	−0.035 (−0.068, −0.003)
Quintiles of Insulin			
Lowest quintile (4.26–49.74 pmol/L)	Reference	Reference	Reference
Q2 (49.80–66.84 pmol/L)	−0.359 (−0.568, −0.149)	−0.359 (−0.570, −0.149)	−0.404 (−0.629, −0.179)
Q3 (66.90–96.36 pmol/L)	−0.378 (−0.593, −0.163)	−0.376 (−0.593, −0.158)	−0.403 (−0.635, −0.170)
Q4 (96.42–935.22 pmol/L)	−0.296 (−0.521, −0.071)	−0.289 (−0.518, −0.060)	−0.256 (−0.506, −0.006)
*p* for trend	0.006	0.009	0.026

Non-adjusted model adjust for: none. Adjust I model adjust for: gender, age, race. Adjust II model adjust for: gender, age, race, education level, income poverty ratio, calcium, glycohemoglobin.

**Table 4:** The association between insulin (10 pmol/L) and spherical equivalent (D) in 2003–2008.

	Non-adjusted, *β* (95% CI)	Adjust I, *β* (95% CI)	Adjust II, *β* (95% CI)
Insulin (10 pmol/L)	−0.006 (−0.019, 0.006)	−0.009 (−0.021, 0.004)	−0.013 (−0.026, 0.001)
Stratified by gender			
Male	−0.011 (−0.028, 0.005)	−0.014 (−0.030, 0.002)	−0.018 (−0.036, −0.000)
Female	−0.000 (−0.018, 0.018)	−0.002 (−0.021, 0.016)	−0.006 (−0.026, 0.013)
Stratified by race			
Mexican American	−0.028 (−0.049, −0.007)	−0.026 (−0.047, −0.005)	−0.021 (−0.043, 0.001)
Other Hispanic	0.006 (−0.037, 0.049)	0.004 (−0.041, 0.049)	−0.006 (−0.051, 0.040)
Non-Hispanic White	−0.013 (−0.039, 0.014)	−0.014 (−0.041, 0.012)	−0.020 (−0.048, 0.008)
Non-Hispanic Black	0.003 (−0.012, 0.017)	0.003 (−0.012, 0.018)	0.001 (−0.017, 0.019)
Other Race	0.051 (−0.044, 0.146)	0.029 (−0.064, 0.122)	0.008 (−0.089, 0.105)
Quintiles of Insulin			
Lowest quintile (4.26–38.34 pmol/L)	Reference	Reference	Reference
Q2 (38.40–59.34 pmol/L)	−0.074 (−0.289, 0.141)	−0.079 (−0.294, 0.136)	−0.082 (−0.304, 0.141)
Q3 (59.40–93.84 pmol/L)	−0.068 (−0.286, 0.150)	−0.093 (−0.314, 0.128)	−0.117 (−0.345, 0.111)
Q4 (93.90–1387.44 pmol/L)	−0.189 (−0.419, 0.042)	−0.235 (−0.469, −0.002)	−0.308 (−0.552, −0.064)
*p* for trend	0.139	0.060	0.017

Non-adjusted model adjust for: none. Adjust I model adjust for: gender, age, race. Adjust II model adjust for: gender, age, race, education level, income poverty ratio, calcium, glycohemoglobin.

**Figure 1:**
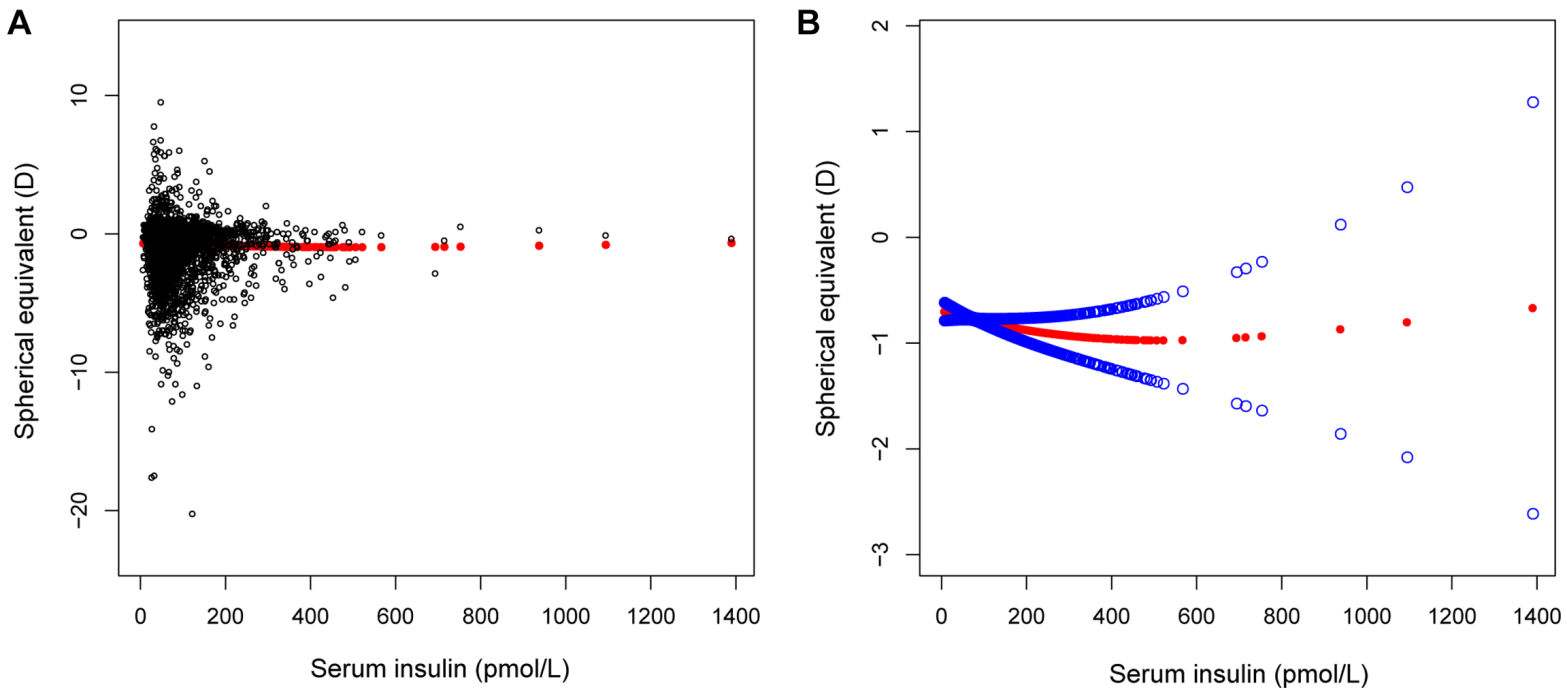
The correlation between serum insulin and spherical equivalent. **(A)** Each sample is represented by a black dot. **(B)** The arc formed by the solid red dots represents the smooth curve fit between the variables. 2 blue bands represent the 95% confidence intervals of the fit results. Adjusted for gender, age, race, education level, income poverty ratio, calcium, glycohemoglobin.

**Figure 2:**
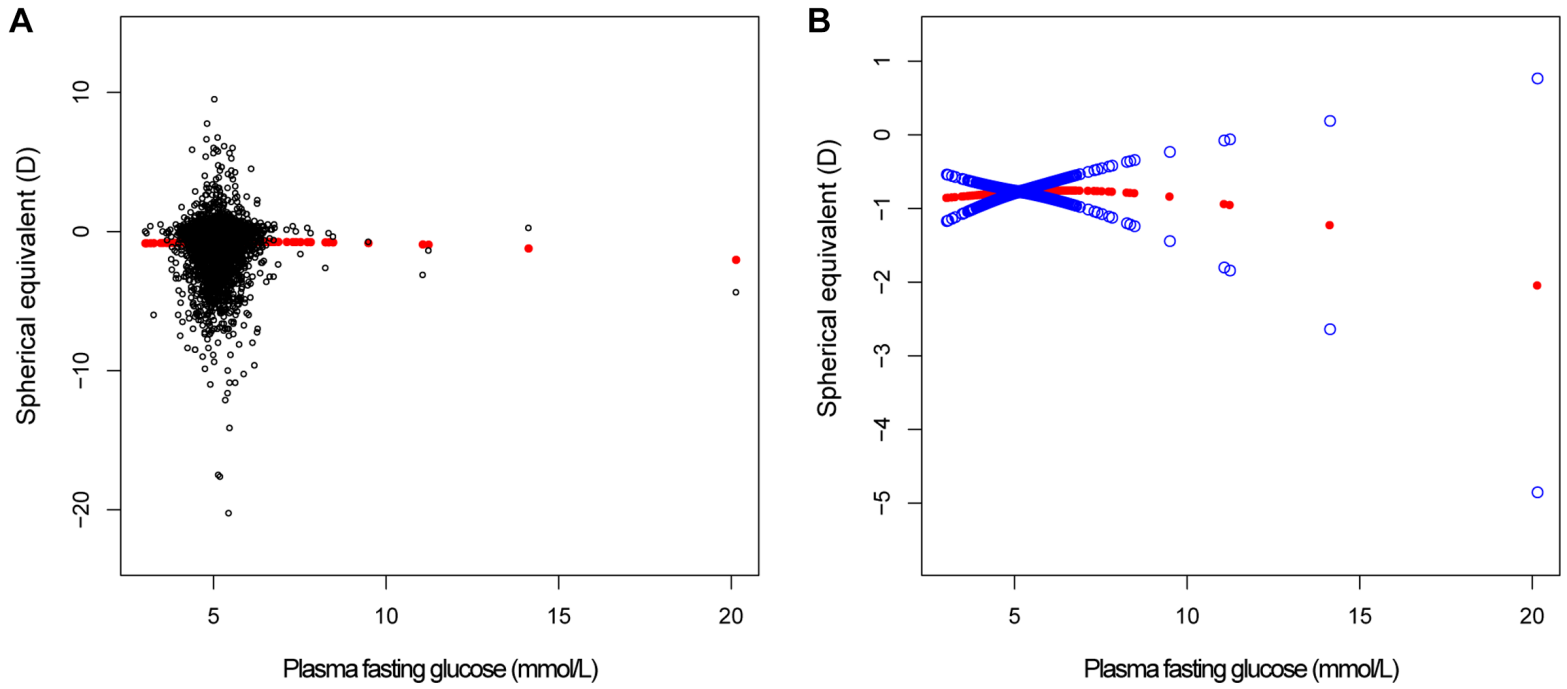
The correlation between plasma fasting glucose and spherical equivalent. **(A)** Each sample is represented by a black dot. **(B)** The arc formed by the solid red dots represents the smooth curve fit between the variables. 2 blue bands represent the 95% confidence intervals of the fit results. Adjusted for gender, age, race, education level, income poverty ratio, calcium, glycohemoglobin.

**Figure 3:**
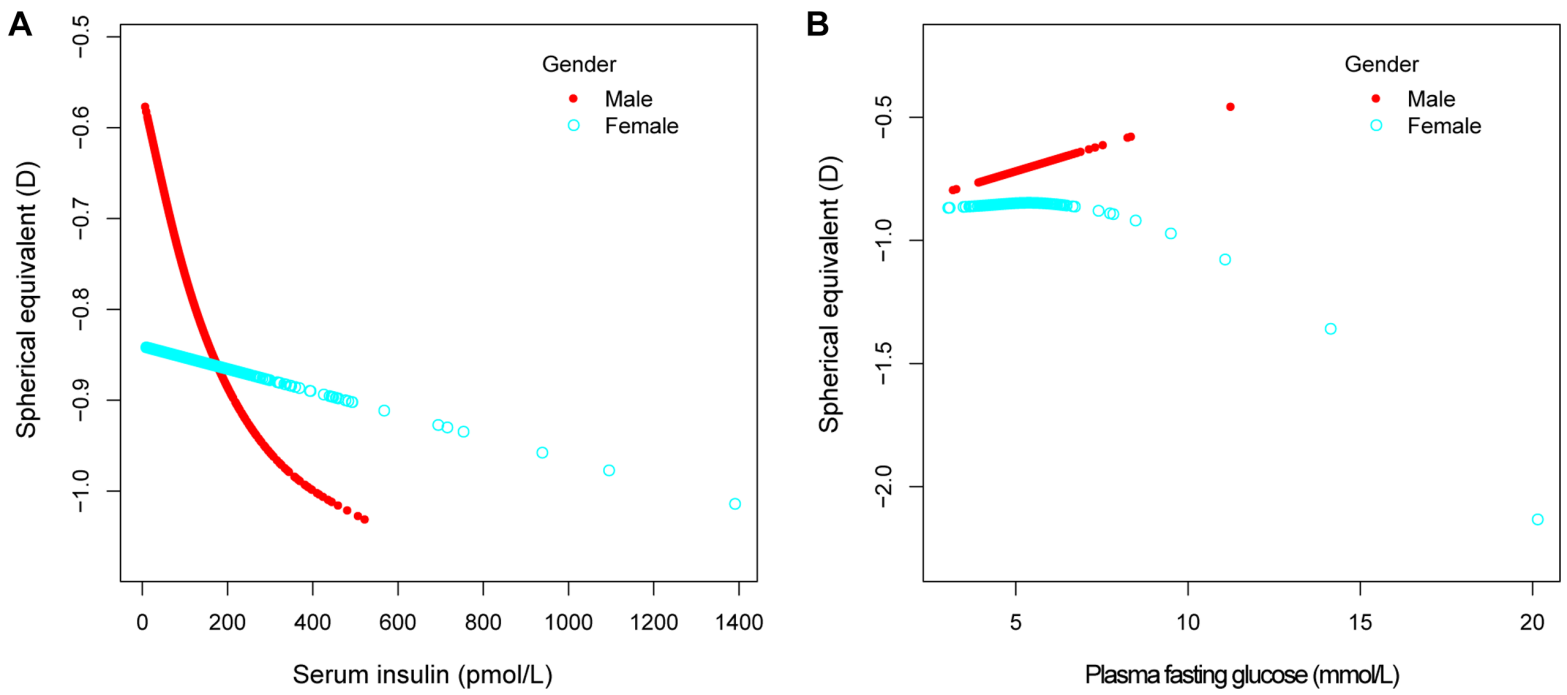
The association between serum insulin, plasma fasting glucose and spherical equivalent, stratified by gender. Age, race, education level, income poverty ratio, calcium, glycohemoglobin were adjusted.

In different multivariable linear regression models, there was no significant correlation between plasma fasting glucose and refractive status (Table 5). Meanwhile, this correlation remained insignificant in the stratified analysis by gender, race, and different concentrations. We also used smoothed curve fitting to reveal the nonlinear relationship between the variables, and the results remained stable (Figures 1–3).

**Table 5:** The association between plasma fasting glucose (mmol/L) and spherical equivalent (D).

	Non-adjusted, *β* (95% CI)	Adjust I, *β* (95% CI)	Adjust II, *β* (95% CI)
Plasma Fasting Glucose (mmol/L)	0.009 (−0.105, 0.122)	−0.033 (−0.150, 0.084)	−0.041 (−0.174, 0.091)
Stratified by gender			
Male	−0.073 (−0.237, 0.091)	−0.085 (−0.251, 0.081)	−0.117 (−0.302, 0.068)
Female	0.022 (−0.142, 0.185)	0.018 (−0.148, 0.184)	0.020 (−0.172, 0.213)
Stratified by race			
Mexican American	−0.120 (−0.259, 0.019)	−0.149 (−0.289, −0.009)	−0.135 (−0.328, 0.058)
Other Hispanic	0.386 (−0.138, 0.910)	0.228 (−0.313, 0.769)	0.217 (−0.387, 0.821)
Non-Hispanic White	−0.029 (−0.290, 0.232)	−0.088 (−0.358, 0.182)	−0.091 (−0.381, 0.199)
Non-Hispanic Black	0.119 (−0.068, 0.307)	0.088 (−0.105, 0.281)	0.090 (−0.125, 0.306)
Other Race	0.227 (−0.219, 0.673)	0.132 (−0.340, 0.603)	0.105 (−0.558, 0.767)
Quintiles of plasma fasting glucose			
Lowest quintile (2.19–4.80 mmol/L)	Reference	Reference	Reference
Q2 (4.81–5.08 mmol/L)	0.067 (−0.094, 0.228)	0.054 (−0.108, 0.215)	0.082 (−0.087, 0.252)
Q3 (5.09–5.37 mmol/L)	0.125 (−0.036, 0.287)	0.077 (−0.088, 0.242)	0.081 (−0.093, 0.254)
Q4 (5.38–38.09 mmol/L)	−0.014 (−0.173, 0.145)	−0.077 (−0.241, 0.088)	−0.082 (−0.259, 0.096)
*p* for trend	0.964	0.375	0.345

Non-adjusted model adjust for: none. Adjust I model adjust for: gender, age, race. Adjust II model adjust for: gender, age, race, education level, income poverty ratio, calcium, glycohemoglobin.

## Discussion

The primary objective of this study was to investigate the potential relationship between serum insulin and plasma fasting glucose levels with refractive status. In the present study, we used a sample of American adolescents aged 12–19 years. The outcome showed a negative association between serum insulin and spherical equivalent in males, and Mexican American, but no significant correlation in female adolescents, non-Hispanic white, non-Hispanic Black, other Hispanic and other races. Subgroup analysis was performed according to insulin measurement and found that the trend of myopia deepening with increasing insulin levels remained robust, especially in male individuals, but there were some differences in the effect between races. Also, among the adolescents included in the analysis demonstrated that fasting glucose levels were not significantly linked to spherical equivalent.

Recent studies have shown that insulin levels may have an effect on refractive status (12, 13). Feldkaemper’s study on chicks discovered that insulin promotes cell proliferation and accelerates the growth of the eye axis (9). As people’s quality of life improves, prolonged high glucose diets lead to more common hyperinsulinemia (14, 15). The chronic plasma hyperinsulin state suppressed hepatic synthesis of insulin–like growth factor binding protein-1 (IGFBP-1), thereby increasing free insulin-like growth factor-1 (IGF-1) and promoting cell and tissue growth (15, 16). Zhuang et al. found that IGF-1 polymorphism was associated with the high myopia population in the Chinese population (17). In addition, reduction of IGFBP-3 could stimulate further tissue growth through its effect on the retinoic acid signaling pathway (18, 19). The results of our study revealed different effects of insulin levels in different races and genders of participants. Individualized monitoring for adolescents may be more beneficial for myopia control and less wasteful of medical resources.

The effect of gender on refractive status is currently more controversial, with some studies finding a higher prevalence of myopia in males (20, 21), and individual studies showing opposite results (22, 23). The results of this study showed a negative correlation between spherical equivalent and insulin levels, and this performance was more significant especially in the male population. Plasma fasting glucose and glycohemoglobin were significantly higher in male individuals than in females in the study population, which may be one of the reasons why men are more susceptible (24). Also, the secretion of sex hormones can lead to differences in the response to insulin between men and women. Some studies have shown that insulin resistance predisposes to the development of myopia (25). Prior to menopause, insulin resistance occurs less frequently in women than in men of the same age. However, this protective effect disappears after menopause, suggesting that estrogen may have some protective effect (26, 27). The above study can partially explain the negative correlation between refractive status and insulin levels in the male population. Because women are less prone to insulin resistance due to the potential protective effect of estrogen, the tendency for myopia is relatively lower.

Chronic high sugar intake can directly affect the body’s plasma glucose, insulin, and glucagon levels (28, 29). Some studies suggest that high plasma glucose levels may be a risk factor for myopia (30, 31). Our multivariable linear regression results, however, showed that elevated fasting glucose levels were not significantly associated with adolescent myopia.

The present research also had certain limitations. To begin with, this research was a cross-sectional study and lacked long-term follow-up data. Second, the measurement of refractive status in adolescents was obtained using a non-ciliary muscle paralysis method, which might have some error. Methodological errors were minimized by fogging the vision and using the median of the three measured refractive values. Due to the limitations of cross-sectional studies, we could only establish correlations and not effectively establish causal relationships. Hence, we adjusted for potential confounding factors in an attempt to minimize this limitation as much as possible.

## Conclusion

Our study found that higher insulin levels were associated with myopia, especially in male adolescents. Refractive status in Mexican Americans showed a negative correlation with rising insulin levels, but this status disappeared after subgroup analysis.

## Data availability statement

The datasets presented in this study can be found in online repositories. The names of the repository/repositories and accession number(s) can be found at: the datasets for this study can be found at www.cdc.gov/nchs/nhanes/.

## Ethics statement

All named authors meet the International Committee of Medical Journal Editors (ICMJE) criteria for authorship for this article, take responsibility for the integrity of the work as a whole, and have given their approval for this version to be published. The NCHS Ethics Review Board granted approval for the conduct of NHANES and written informed consents were obtained from all participants. The studies were conducted in accordance with the local legislation and institutional requirements. Written informed consent for participation in this study was provided by the participants' legal guardians/next of kin.

## Author contributions

PH: Data curation, Formal analysis, Writing – original draft, Writing – review & editing. JL: Conceptualization, Data curation, Writing – original draft. MH: Writing – original draft. YF: Writing – original draft. MW: Conceptualization, Writing – review & editing.

## Footnotes

^1^http://cdc.gov/nchs/nhanes

^2^http://www.R-project.org

^3^http://www.empowerstats.com
